# Soil qualities and change rules of *Eucalyptus grandis* × *Eucalyptus urophylla* plantation with different slash disposals

**DOI:** 10.1038/s41598-022-25687-0

**Published:** 2022-12-05

**Authors:** Lin Zhang, Zongfu Chen, Qinzhan Wu, Kangting Huang, Jianke Wen, Hui Li, Lingyue Zhu, Yabin Tang, Lijun Chen, Lichao Wu

**Affiliations:** 1 Key Laboratory of Soil and Water Conservation and Desertification Combating of Hunan Province, College of Forestry, Central South University of Forestry and Technology, Changsha, Hunan China; 2 State-Owned Daguishan Forest Farm, Hezhou, Guangxi China; 3 Guangxi Lvtuo Forestry CO., LTD, Nanning, Guangxi China; 4 Guangxi Diyuan Zhiben Fertilizer Co., Ltd, Nanning, Guangxi China; 5 Key Laboratory of Cultivation and Protection for Non‐Wood Forest Trees of National Ministry of Education, Central South University of Forestry and Technology, Changsha, Hunan China

**Keywords:** Forest ecology, Forestry

## Abstract

Slash disposal changes soil quality by affecting soil properties and nutrient cycling, and the appropriate disposal approaches remain controversial. This work aimed to explore the impact of different slash disposal methods on soil qualities. For this purpose, a *Eucalyptus grandis* × *Eucalyptus urophylla* plantation that had been cultivated in 2002 and felled for the third time in 2016 was established in Hezhou City, China. Burning forest (BF, for moderate intensity fire) and no-burning forest (NF) were set in the plantation, and the native evergreen broadleaf forest near the plantation was used as the control (CK). Soils were sampled quarterly in 2017, and 27 indicators that represent soil physical, chemical, and biological properties were analyzed and compared through the analysis of the sustainability index (SI), which adopts five indices to calculate soil quality. The obtained data showed that the indicators of BF and NF, except for the total potassium content, were much lower than those of CK. The physical properties (Max-WHC, CWHC, Min-WHC, MMC, CPD, TPD) of NF were significantly better (29.07%, 30.98%, 29.61%, 52.08%, 21.89%, 19.76%) than those of BF, unlike the chemical properties of BF (SOM, TN, ACa, AFe, AMn, ACu, AZn) were significantly better than those of NF (45.61%, 81.33%, 12.78%, 23.18%, 96.13%, 144.30%, 114.04%). The enzymatic activities of NF (URE, APHO) were significantly better (43.33%, 156.58%)than those of BF, except the activities of INV (− 25.21%). Results of SI showed that the soil quality of CK was much better than that of BF, and NF the worst. But it exhibited the most unevenness of CK, followed by NF, and BF the best. The change rules of BF and NF were contrasting, and soil quality reached the same level after half a year. In summary, the soil qualities, either BF or CK, were not comparable to that of CK. BF increased the soil quality fleetly and transiently, and NF was sustainable for the eucalyptus plantation.

## Introduction

Since the official signing of the Paris Climate Agreement in 2015, the concept of carbon neutrality has been gaining further popularity, and most countries and regions around the world have given specific timetables for achieving carbon neutrality. Energy conservation and afforestation are two ways to achieve carbon neutrality, and the sustainable development of plantations is the main means of afforestation^1^. Although large-scale eucalyptus plantations provide massive economic and social benefits^2^, their high-intensity operation has caused problems, such as soil structure destruction and soil fertility decline in plantations^3^ and large amount of CO_2_ emission due to the burning forest as the method of slash disposal. No-burning forest is an effective way to address the ecological imbalance and improve the soil quality of plantations, and it can save plantations from fire, improve plantation ecology, and effectively slow down the decline of plantation soil quality^4^, and definitely greatly reduce carbon emission because of the avoidance of the fire when disposal the slash. Therefore, studying soil quality under no-burning forest conditions is of practical importance because it helps guide the production practices of plantations and reduce carbon emission and achieve ecological management and sustainable development of plantations.

Burning forest is currently the most extensively adopted way of slash disposal in eucalyptus plantations. With BF, the slashes were burn up with the supervision after they were completely dry under natural condition. It has many advantages, but it is also a critical contributor to the decline of the soil quality of eucalyptus plantations^5^. Burning forest reduces the accumulation of phenolic substances and the risk of chemosensory and autotoxic effects of plants by burning out apoplastic materials, logging residues, and ground grasses. Furthermore, it stimulates the growth of saplings by killing pests and releasing a large amount of nutrients while burning^6^. However, its disadvantages are also obvious. The fire removes all combustibles from the surface of the soil, resulting in long-term exposure of the soil surface, which causes soil erosion, massive losses of soil nutrients, and the eventual decline of soil quality^7^. Different temperatures caused by different intensity of fire will mutually converse the soil between clayey and sandy. When the temperature reaches 200 °C, the fire will cause the fragmentation of soil aggregates and sands, which accelerated the rate of soil weathering^8^; When the temperature was higher than 800 °C, anhydrous minerals were formed^9^, and the smolder at the tree-root and slash-stack generated ultra-high temperature, that can melt soil mineral particles, making them change from clayey to silty^10^.When burning forest, the high temperature of the fire killed the microorganisms living in the soil surface and humus layer, and the organic carbon, nitrogen and other nutrient elements were lost in the form of gas and water solution. Meanwhile, the fire caused the death of the vegetation roots, making the mycorrhizal fungi lose their hosts, those were the reasons that the structure and function of the soil microbial community were significantly changed by burning forest. The fire also killed animals such as soil nematodes and forest animals. If the soil animals were deep in the soil, they might survive, while the forest animals were basically exterminated, therefore, large-scale burning led to the reduction of forest biodiversity, which could not be recovered in a short time. All in all, the BF destroys the soil structure^11^, reduces the water-holding and nutrient capacity of the soil^12^, threatens the survival of soil animals, and alters the soil microbial community structure^13^, resulting in ecological imbalance^14^, biodiversity reduction^15^, and reduced soil quality.

No-burning forest reduces the risk of fire, carbon dioxide emission, soot, and soil erosion and is therefore considered an effective way to slow down the decline of soil quality and increase biodiversity to achieve the sustainable development of eucalyptus plantations^16^. However, it also suffers from high production costs, slow pre-crop growth, and severe pests and diseases. Slashes are a critical reason for these problems. On the one hand, the roots of the original vegetation are not destroyed in no-burning forests^17^, which increases the cost of clearing and nurturing the plantation. On the other hand, the hot and humid environment created by the decomposition process of slashes is likely to breed diseases and insects, which are harmful to the seedlings^18^. The slow decomposition rate of slashes in the early stage that makes the soil nutrients insufficient coupled with the rapid growth of ground cover and weeds, which compete with seedlings for water and fertilizer, limits the growth of the saplings^19^.

Different slash disposal methods change the decomposition rate of slashes and considerably affect soil quality and sapling growth, but no uniform conclusion has been established about which method produces the best results. Burning, piling^20^, spreading, and shredding^21^ are several methods of slash disposal that have been commonly studied. Chen Mengdi^22^ compared soil qualities under three methods of slash disposal and concluded that soil quality is the best in artificially controlled burning, and the time and intensity of the fire are the decisive factors^23^. Yu YangYang^24^ compared the effects of five methods of slash disposal and concluded that leaving slashes in situ is more beneficial to the growth of eucalyptus plantations than other approaches. Lee ChangJun^25^ concluded that the addition or removal of slashes exerts some effects on soil carbon^26^, but Bushra^14^ posited that this effect is not significant within two years. The reason for these controversies might be that researchers always choose to evaluate soil quality in the beginning and end of their study and ignore the fact that the decomposition of slashes is unstable during the study period because it is affected by climate, rainfall, temperature, and human disturbance, which affect the activity of microbial thus change the decomposition rate, cause soil quality to change nonlinearly. Therefore, studying the change rule of soil quality is crucial for reasonable nurturing, timely fertilizer supplementation, and precise fertilizer application in planting practice.

Many methods, such as hierarchical analysis^27^, life cycle method^28^, dynamic evaluation method, perspective method^29^, and a combination of GIS, fuzzy mathematics, and minimum data set comprehensive index method^30–32^, can be used to assess soil quality. The sustainability index^33^ categorizes soil indicators into several soil indices representing soil capacities on the basis of existing experience and expresses soil quality through the area of a polygon drawn with the soil index values overlapping in the first part, extending outward at the same angle of entrenchment, and connected at the end. A large polygon area denotes good soil quality. The sustainability index provides an intuitive picture of the difference in the quality of different soils and the causes of the difference in soil quality from the area and shape of polygons compared with the composite index; hence, it was used in this study.

By referring to existing studies, we propose the following scientific hypotheses. (1) In terms of soil chemical indicators, the soil physical and enzyme activity indicators of no-burning forest (NF) are better than those of burning forest (BF), and NF is better in water, gas, and microbial environment provision but poorer in fertilizer provision. (2) The soil quality of BF is generally higher than that of NF during the study, and their change rules differ. The soil quality of NF increases rapidly then decreases slowly, whereas that of BF continuously and unsteadily decreases.

To prove our hypotheses, we set up a field experiment in a Eucalyptus grandis × Eucalyptus urophylla (*E. grandis* × *E. urophylla*) plantation, which was cultivated continuously for 15 years in the East Branch of Guangxi State-owned Daguishan Forest Farm (GSDFF). A total of 27 soil indicators were sampled and analyzed for four seasons over one year. The sustainability index was applied to assess the soil quality and change rules. The main objectives of this study were (1) to explore how the different ways of slash disposal affect soil physical, chemical, enzyme properties and the capacity for water, fertilizer, air, and microbial environment provision to the soil and (2) to employ SI in assessing the soil quality and change rules of different ways of slash disposal. The study revealed the effects of slash disposal on the eucalyptus plantation. The results help in selecting appropriate treatments and provide theoretical management insights into applying fertilizers precisely and measurably, thus facilitating the sustainable development of plantations.

## Results

### Difference in soil physical properties

The physical indicators differed significantly among the three treatments. The Maximum Water-Holding Capacity (Max-WHC), Minimum Water-holding Capacity (Min-WHC), Capillary Water-Holding Capacity (CWHC, and MMC) of NF were higher than those of BF, and most of the indicators of the two were lower than those of CK (Table 1, p < 0.05). NF significantly increased Max-WHC (29.07%), Min-WHC (30.98%), CWHC (29.61%), and MMC (52.08%) compared with BF and made these indicators close to the value for CK. No differences in Non-capillary Porosity Degree (NPD), Capillary Porosity Degree (CPD), Total Porosity Degree (TPD), and Ventilation Degree (VD) were observed between BF and NF. NF increased CPD (21.88%) and TPD (19.76%) to a certain extent, making the two indicators close to the value for CK, unlike the significantly lower indicators of BF compared with CK (CPD 42.84%, TPD 45.14%). No significant differences in the indicators of Volumetric Moisture Content (VMC) and Bulk Density (BD) were observed among the three treatments. In addition, the soil physical properties of CK were the best, followed by those of NF, and BF the worst.

**Table 1:** ANOVA of the soil physical properties of different slash disposal methods in GSDFF.

Indicator	BF (n = 12)	NF (n = 12)	CK (n = 12)	*p*
Max-WHC (g/kg)	404.88 ± 59.74^b^	522.56 ± 88.85^a^	562.76 ± 92.33^a^	< 0.001
CWHC (g/kg)	372.56 ± 52.49^b^	487.99 ± 99.11^a^	494.12 ± 104.87^a^	0.003
Min-WHC (g/kg)	364.88 ± 49.85^b^	472.93 ± 100.48^a^	486.03 ± 109.6^a^	0.004
BD (g cm^−3^)	1.05 ± 0.1	0.96 ± 0.14	1.16 ± 0.38	0.149
MMC (g kg^−1^)	283.15 ± 75.09^b^	430.62 ± 96.66^a^	392.24 ± 112.31^a^	0.002
VMC (gl^−1^)	299.25 ± 69.38	415.28 ± 104.9	430.52 ± 195.63	0.044
NPD (%)	3.76 ± 1.89^b^	3.23 ± 1.1^b^	10.01 ± 4.2^a^	< 0.001
CPD (%)	38.47 ± 5.55^b^	46.89 ± 10.27^ab^	54.95 ± 22.06^a^	0.03
TPD (%)	41.85 ± 6.5^b^	50.12 ± 9.41^ab^	60.74 ± 28.71^a^	0.046
VD (%)	10.93 ± 2.04^b^	8.6 ± 2.49^b^	22.47 ± 7.76^a^	< 0.001

The data form as: average value ± standard deviation standard of significant difference. The relationships of superscript letters a, b and c are that a is significantly higher than b and c, and b is significantly higher than c.

### Difference in soil chemical properties

The physical indicators differed significantly among the three treatments. The Soil Organic Matter (SOM), Total Nitrogen (TN), Available Ferri (AFe), Available Manganese (AMn), Available Zinc (AZn), and Available Copper (ACu) of NF were higher than those of BF, and most of the indicators of the two were lower than the value for CK (Table 2, p < 0.05). NF significantly decreased SOM (31.32%), TN (44.85%), AFe (18.81%), AMn (49.01%), AZn (53.28%), ACu (59.07%), and pH (5.67%) and increased Available Phosphorus (AP, 143.48%) compared with BF. The AP of NF was significantly higher (55.56%) than that of CK. NF and BF significantly increased Total Potassium (TK, 37.82%, 42.13%), and no significant difference in Available Potassium (AK) was observed compared with CK. The Available Calcium (ACa) of BF was significantly higher than that of CK (24.59%), and Available Magnesium (AMg) and ACu showed no significant difference in the three treatments. The chemical properties of CK were the best, followed by those of BF, and NF the worst.

**Table 2:** ANOVA of the soil chemical properties of different slash disposal methods in GSDFF.

Indicator	BF (n = 12)	NF (n = 12)	CK (n = 12)	*p*
SOM (g kg^−1^)	34.32 ± 7.85^b^	23.57 ± 1.47^c^	44.84 ± 5.85^a^	< 0.001
pH	4.41 ± 0.26^a^	4.16 ± 0.03^b^	4.34 ± 0.19^a^	0.008
TN (g kg^−1^)	2.72 ± 0.42^b^	1.5 ± 0.2^c^	3.16 ± 0.54^a^	< 0.001
TP (g kg^−1^)	0.34 ± 0.07^b^	0.34 ± 0.03^b^	0.49 ± 0.07^a^	< 0.001
TK (g kg^−1^)	35.02 ± 6.99^a^	33.96 ± 2.75^a^	24.64 ± 8.24^b^	0.001
AP (mg kg^−1^)	0.92 ± 0.65^c^	2.24 ± 0.24^a^	1.44 ± 0.47^b^	< 0.001
AK (mg kg^−1^)	108.03 ± 44.3	109.45 ± 6.98	90.33 ± 47.51	0.394
ACa (mg kg^−1^)	87.26 ± 8.27^a^	77.37 ± 11.86^ab^	70.04 ± 12.47^b^	0.002
AMg (mg kg^−1^)	19.83 ± 5.03	17 ± 3.39	20.28 ± 6.19	0.235
AFe (mg kg^−1^)	420.84 ± 91.13^b^	341.66 ± 41.61^c^	534.45 ± 86.79^a^	< 0.001
AMn (mg kg^−1^)	9.63 ± 5.47^b^	4.91 ± 1.41^c^	14.21 ± 5.5^a^	< 0.001
ACu (mg kg^−1^)	1.93 ± 0.48^a^	0.79 ± 0.08^b^	1.78 ± 0.81^a^	< 0.001
AZn (mg kg^−1^)	3.81 ± 1.73^b^	1.78 ± 0.07^c^	5.31 ± 0.91^a^	< 0.001

### Difference in soil enzymatic properties

Significant differences were observed in enzyme activities among the three treatments. The enzyme responses to different activities varied in the treatments, and the enzyme activity of NF was lower than that of BF (Table 3, p < 0.05). NF significantly decreased urease (URE) (30.23%) and aid phoatase (APHO) (61.03%) and increased invertase (INV) (25.21%). Catalase (CAT) had no significant difference compared with that for BF. The INV of NF and the APHO of BF were significantly increased by 28.76% and 24.97%, respectively, and the others were significantly decreased compared with CK.

**Table 3:** ANOVA of soil enzymatic activities of different slash disposals in GSDFF.

Indicator	BF (n = 12)	NF (n = 12)	CK (n = 12)	*p*
URE (mg/g)	0.43 ± 0.05^b^	0.3 ± 0.05^c^	0.63 ± 0.07^a^	< 0.001
APHO (mg/g)	9.16 ± 1.65^a^	3.57 ± 1.21^c^	7.33 ± 1.02^b^	< 0.001
INV (mg/g)	21.06 ± 1.92^b^	26.37 ± 1.27^a^	20.48 ± 6.66^b^	0.002
CAT (mg/g)	2.23 ± 0.37^b^	2.14 ± 0.3^b^	2.46 ± 0.17^a^	0.003

### Difference in change rules

**Figure 1:**
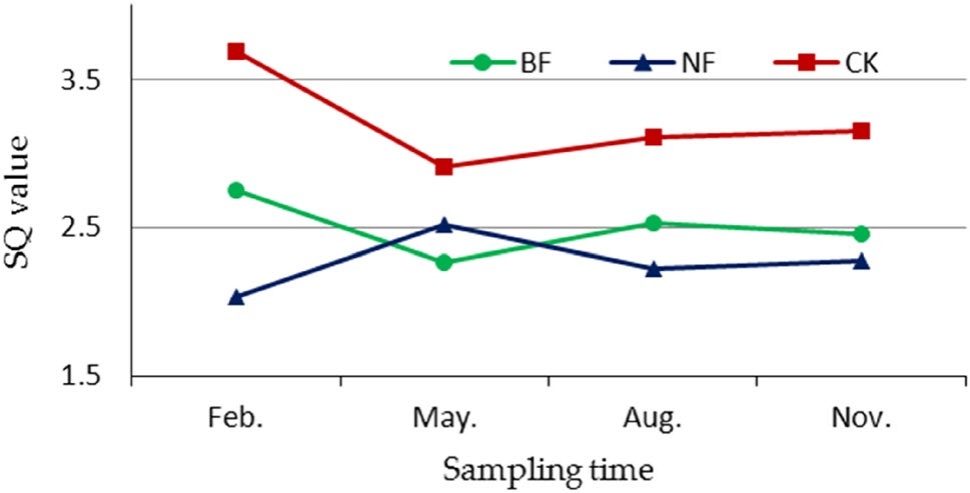
Change rules of different slash disposals in GSDFF. BF means burning forest, NF means no-burning forest, CK means control.

The soil quality of BF was significantly lower than that of CK, and the change rule was similar to that of CK and opposite to that of NF (Fig. 1). The soil quality of NF (1.57) was significantly lower than that of BF (2.07) and CK (3.69) in Feb., and NF (2.30) was significantly higher than BF (1.94) in May. From Feb. to May, NF increased by 23.56%, and BF and CK decreased by 17.61% and 21.22%, respectively. From May to Aug., BF increased to 2.18 and CK increased to 3.11, but NF decreased to 1.89. Until Aug., BF and CK showed the same change rules, but NF was opposite to the two. From Aug. to Nov., BF slightly decreased to 2.08, and NF and CK slightly increased to 1.95 and 3.15, respectively. BF and CK had the same change rules, that is, they achieved the highest in Feb. and significantly decreased from Feb. to May. NF showed the opposite trend.

### Difference in soil qualities

**Figure 2:**
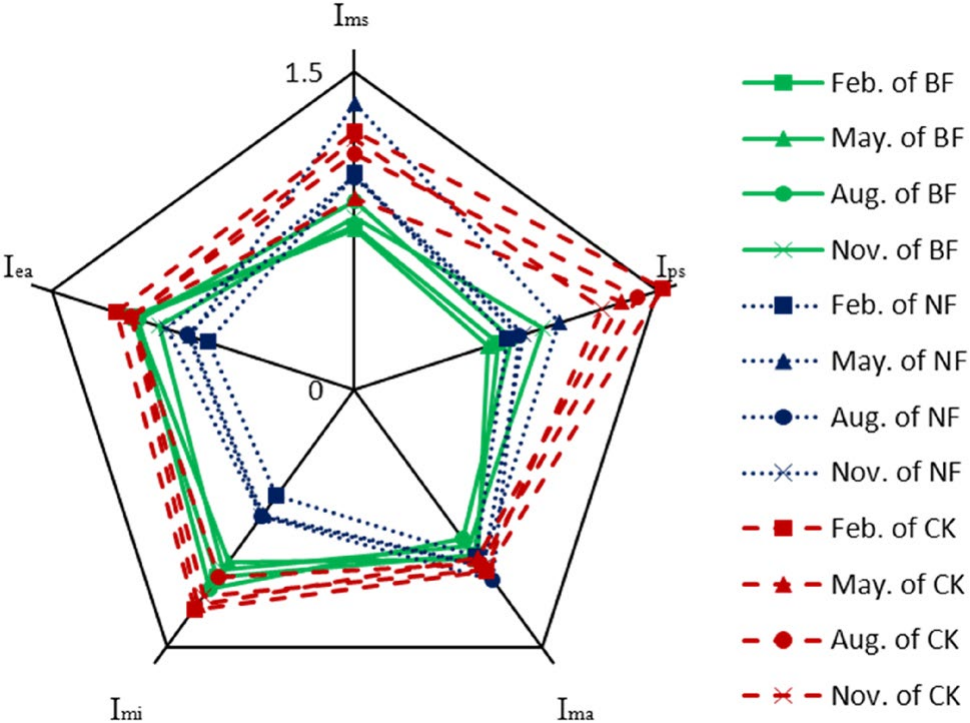
Soil qualities of different slash disposals in GSDFF. BF means burning forest, NF means no-burning forest, CK means the control. I_ms_ means the moisture index, I_ps_ means the porosity index, I_ma_ means the macro-element index, I_mi_ means the minor element index, I_ea_ means the enzyme activity index.

No significant differences in soil qualities (pentagon area) were observed, but the soil indices differed between BF and NF; the two had significantly lower soil qualities and higher *C*_*u*_ than CK (Fig. 2). No significant differences were observed between BF (2.07) and NF (1.93), whose soil qualities were significantly lower than that of CK (3.22). NF decreased I_ea_ (22.04%) and I_mi_ (34.32%) and increased I_ms_ (35.70%) compared with BF. *C*_*uBF*_ (0.14) < *C*_*uNF*_ (0.17) < *C*_*uCK*_ (0.20) was calculated based on *Eq. *(3). The high I_ps_ and low I_ms_ were the reasons for the high value of *C*_*uCK*_, which was in line with the situation that the soil structure was good and soil fertility was poor in the local primary forest. BF destroyed the soil structure of the primary forest [43], which decreased I_ps_ and effectively supplied soil nutrients. The accumulated organic matter improved the soil enzyme activities, which increased I_ea_ and I_mi_. These were the reasons for the unevenness of the soil indices, and the results of NF were opposite to those of BF.

## Discussion

### Effects of different slash disposals on soil quality evaluated by soil indicators

We found that the Max-WHC, Min-WHC, CWHC, and MMC of NF were higher than those of BF, suggesting that BF significantly reduced the water-holding properties. BF hardened the topsoil, thus forming a waterproof layer that prevented water infiltration^34^, and the exposed topsoil was susceptible to hydraulic erosion that reduced the water-holding properties^35^. Meanwhile, the slashes covered on the topsoil of NF could reduce rain erosion, increase infiltration and water storage, and significantly improve water-holding properties^36^. No differences in NPD, CPD, TPD, VD, and BD were observed between BF and NF, but the CPD and TPD of BF were significantly lower than those of CK, indicating that NF could maintain the aeration properties of soil and BF could not maintain them. Soil water-holding and aeration properties comprehensively reflect the soil structure^37^. Therefore, NF helped maintain the soil structure to a certain extent.

The obtained data showed that SOM, TN, AFe, AMn, AZn, and ACu in NF were significantly lower than those in BF (31.32%, 44.85%, 18.81%, 49.01%, 53.28%, and 59.07%, respectively), indicating that BF significantly increased the soil nutrients, especially the content of trace elements, and those won’t change until two years later^14^. TN is an important indicator to evaluate soil quality, and NF is generally believed to increase TN^38^. This result is contrary to the result of this study possibly because the growth of shrubs and grass of NF was prosperous, which consumed the mass of N element. The decomposition of the slashes of NF released acidic substances, led to soil acidification^39^, and a significant decrease in pH, which significantly reduced the trace elements^40^. The pH of BF was slightly higher than that of CK, which was probably due to the volatilization of phenolic acids^41^ and other acidic substances in the slashes caused by fire^42^. Notably, BF can reduce the allelopathy of *E. urophylla* plantations and help maintain biodiversity. The generation of AP is affected by SOM, pH, APHO, and some organic acids secreted by plant roots^43^, so BF is conducive to the release of the P element, which is inconsistent with the result of the current study that the AP of NF was significantly higher than that of CK and BF; this finding needs further exploration. SOM can comprehensively reflect soil physical and chemical properties^44^, and as indicated by the lack of any significant difference in BD, NF significantly reduced the soil chemical properties.

The significant differences in soil enzyme activities under the different treatments indicated that soil enzyme activities were sensitive to fire reactions^45^. The URE of NF was significantly lower than that of BF, which might be a critical reason why the TN of NF was significantly lower than that of BF. The APHO of BF increased by 24.97% relative to that of CK, indicating that the activity of the phosphate solubilizing microorganisms was positively affected. The CAT of NF was significantly higher than that of BF and CK and was more favorable for the growth of plants^46^, all of which were possibly affected by the increase in soil moisture content and decrease in pH^47^. The results were inconsistent with our prediction of soil enzyme activity perhaps because the effect of fire intensity was not considered. The activity of microorganisms is sensitive to temperatures at 0–10 cm depth in the soil^48^. The moderate intensity of the fire that we used may have failed to affect the activity of microorganisms at 10–20 cm depth in the 0–20 cm soil that we sampled.

### Effect of different slash disposals on soil qualities and change rules assessed by SI

No differences were observed between BF (2.07) and NF (1.93) in soil quality (pentagon area), and CK (3.21) was significantly higher than the two (Fig. 2). BF had the best evenness of soil indices, and CK had the worst. The air provision capacity was significantly better, and the fertilizer provision capacity was significantly worse than the other capacities within CK, which caused the maximum *C*_*u*_ of the three and truly reflected the local soil conditions. BF significantly upgraded the fertilizer provision and microbial survival capacities and downgraded the water provision capacity because the fire destroyed the soil structure, which led to the minimum *C*_*u*_. With BF can’t maintain the high fertilizer provision^6^ and can’t regain the soil structure it destroyed^49^, this trend would continue in a long term. According to the analysis of the soil indicators, the improvement of the fertilizer provision capacity by BF was mainly due to the increase in soil trace element content^50^, and this result is consistent with that of Zhou Zhili^51^. NF maintained the soil structure effectively but supplied the consumption of medium and trace elements in *E. grandis* × *E. urophylla* plantation insufficiently, resulting in lower I_mi_ and higher *C*_*u*_ than BF.

Significant differences in soil quality were observed between BF and NF within half a year. The reason was that the rate of nutrient release from the slashes and the plant–soil interaction varied. In February(Feb.), BF generated a mass of nutrients, which greatly stimulated the growth of eucalyptus^52^. However, the nutrient supplement was insufficient in the follow-up, which reduced the soil quality rapidly (May). The decomposition of the slashes of NF was slow in Feb., so the nutrients were insufficient, leading to low soil quality and restricted growth of eucalyptus. However, with the rapid decomposition of slashes in May^53^, the soil quality increased substantially, and with the decomposition entering a slow and long-term end after that, the soil quality stabilized.

### Implications for management

The soil qualities between BF and NF were significant different within half a year, and tends to be consistent after half a year, but NF had better soil structure and water-holding properties, and its soil quality increased at the end of the study, which is favorable for soil and water conservation and sustainable for the plantation. At the early stage of NF (Fig. 1, Feb.), fertilizers should be fully applied to increase the soil nutrient content, and herbicides should be used to inhibit the growth of shrubs and grasses. In April (Fig. 1, May), eucalyptus reached its growth peak of the year, which coincidentally was the time when the slash decomposition was the fastest. Attention should be paid to the required nutrients and fertilizer, and N is the most needed element. Most studies have found a significant negative correlation between pH and trace elements^40,54,55^. We noted that these studies were based on alkaline soils, which are contrary to the acidic soils we studied. From the perspective of acid–base balance, a low pH in acidic soils would lead to the lack of trace elements in soils. Therefore, pH can be enhanced by using appropriate alkaline substances, such as quicklime and alkaline residue. The application of organic fertilizer in Feb. would change the decomposition of slashes, which might lead to the following problems. First, this application is out of sync with the growth of eucalyptus^56^, leading to the overgrowth of shrubs and other plants and resulting in fertilizer wastage. Second, it might lead to severe soil acidification and high soil temperature^57^, which are not conducive to sapling survival. In addition, the decomposition of the slashes in this work produced a mass of organic fertilizer in May. Therefore, we suggest that chemical fertilizer should be applied in Feb. and May as required, and organic fertilizer should not be applied until Aug.

## Methods

### Experimental site and soil characteristics

The experimental site is located in GSDFF, Hezhou City, China (24°7′4″–24°7′18″N and 111°42′59″–111°43′7″E). The area is at the boundary of the middle and south subtropical parts and has a subtropical humid monsoon climate with an annual mean rainfall of 2056 mm. The annual mean temperature is 19.3 °C, the maximum temperature is 39.7 °C, the minimum temperature is − 2.4 °C, and the annual accumulated temperature is 6243 °C. The average annual rainfall is 2056 mm, and the annual evaporation is 1275 mm. The average relative humidity is 82%, the annual average number of sunshine hours is 1600–1800, and the frost season lasts for 12 days. The experimental sites are located in hilly areas with altitudes ranging within 200–300 m.

The area of the experiment point is 330 hm^2^, and the altitude is 250–280 m. The soil type of the study area is alliticudic ferrosols, which was derived from Cambrian sand-stone according to the Chinese Soil Taxonomic Classification. The thickness of the soil is approximately 80 cm. The *E. grandis* × *E. urophylla* plantation was established based on the local evergreen broad-leaved forest in 2002, first felled in 2007, reestablished by sprout, and felled for the second time in 2012. Different slash disposal ways were set for reforestation after the third felling in Dec. 2016. *E. grandis* × *E. urophylla* (DH32-29) was planted with a row spacing of 2 m × 3 m and a planting density of 1665 plants hm^−2^. A compound fertilizer (nitrogen: phosphorus: potassium) with a ratio of 15:6:9 was applied. The base fertilizer was applied at 832.5 kg hm^−2^ (complex fertilizer) while planting, and the topdressing was applied at 832.5 kg hm^−2^for the next three seasons.

### Experimental site and soil characteristics

In December 2016, a 13.9 hm^−2^ section with a similar altitude, soil, climate, and other factors was selected in the above-mentioned plantation. Three blocks were arranged based on the random block design, and each block was set with two treatments of random block arrangement. For the first treatment (BF), moderate-intensity fire treatment was carried out on the natural, fully dried slashes. For the second treatment (NF), the slashes were left in situ according to the natural state. Each treatment plot was sized 1 ha in the 13.9 ha section, and a standard plot of 20 × 20 m was set in the center. The adjacent primary evergreen broad-leaved forest was selected as the control (CK).

### Soil sampling

Soils were sampled in February (Feb.), May, August (Aug.), and November (Nov.) in 2017. Topsoil samples (0–20 cm) were collected from the standard plots by using a ring knife and the cloth bag method. Three soil samples were collected from each standard plot with a ring knife (100 cm^3^) and taken to the laboratory for the determination of soil physical properties. After removing humus and impurities from the soil surface, a manual carbon steel auger (inner diameter 5 cm, height 20 cm) was utilized to take 6–8 soil samples according to the “s” type. Then, the soil samples were mixed, quartered individually, and placed in a cloth bag (1000 g of each sample). The bag was taken to the laboratory and placed on a drying plate. The samples were spread into a thin layer of 2–3 cm and dried naturally at room temperature without direct sunlight and exposure. When the soil samples were half dry, the large soil particles were crushed by hand. The soil samples were ground when they were completely dry; sieved with 2, 1, and 0.149 mm meshes; and placed in Ziplock bags separately. They were used to measure soil chemical properties and soil enzyme activities.

### Determination of soil physical and chemical properties

A cutting ring^58^ was used to determine the soil maximum water-holding capacity (Max-WHC), soil minimum water-holding capacity (Min-WHC), soil capillary water-holding capacity (CWHC), soil mass moisture content (MMC), soil volumetric moisture content (VMC), soil non-capillary porosity degree (NPD), soil capillary porosity degree (CPD), soil total porosity degree (TPD), soil ventilation degree (VD), and soil bulk density (BD). Soil pH was determined with a pH meter at a soil/water ratio of 1:2.5^59^. The Kjeldahl method^60^ was used to determine the total nitrogen content (TN). Total phosphorus (TP) and available phosphorus (AP) were measured with digestion^61^ and Mehlich 3 methods^62^, respectively, by using a Smartchem 200 Discrete Chemistry An-alyzer (West Co. Scientific Instruments, Brookfield, CT, USA). Total potassium (TK) and available potassium (AK) were measured with digestion^63^ and Mehlich 3 methods, respectively^64^, by using a flame photometric detector. Available calcium (ACa), available magnesium (AMn), available iron (AFe), available manganese (AMn), available copper (ACu), and available zinc (AZn) were measured with the Mehlich 3 method and atomic absorption spectrophotometry. Soil organic matter (SOM) was determined by dichromate wet combustion and visible spectrophotometry.

### Determination of soil enzymatic activities

Soil urease activity (URE) was determined with the colorimetric method by using sodium phenol and sodium hypochlorite^65^. Airdried soil (5 g) was evenly mixed with 1 mL of toluene for 15 min. Urea was used as a substrate for urease determination. Soil catalase activity (CAT) was estimated with the Johnson–Temple method by utilizing 1 g of air-dried soil titrated with 0.1 mol/L KMnO_4_. The volume of each titration was measured, and the activity was calculated^65^. Acid phosphatase (APHO) was determined via the colorimetric method by using disodium phenyl phosphate^65^. Soil invertase activity was determined with the 3,5-dinitrosalicylic acid method^66^ with a sucrose solution as a substrate^67^.

### Sustainability index (SI)

The composite index is the most mature and widely used approach for soil quality^68^, and the SI provides a more intuitive picture of the difference in the quality of different soils compared with the composite index, and shows the causes of the difference in soil quality from the shape of polygons, that the composite index can’t afford. Soil quality is considered to be the capacity of soil to provide water, fertilizer, air, and heat^69^. Therefore, in this study, the indicators Max-WHC, Min-WHC, CWHC, and VMC were classified as the moisture index (I_ms_) to represent the water-provision capacity. NPD, CPD, and TPD were classified as the porosity index (I_ps_) to represent the air-provision capacity. and I_ms_ and I_ps_ can measure the soil structure^70^. TN, TP, TK, AP, and AK were classified as the macro-element index (I_ma_), and ACa, AMg, AFe, AMn, Acu, and AZn were classified as the minor element index (I_mi_). I_ma_ and I_mi_ can measure the fertilizer-provision capacity. URE, CAT, APHO, and INV were classified as the enzyme activity index (I_ea_), which was used to represent the microbial survival capacity^71^. The five soil indices were calculated with Eq. (1), and soil quality was calculated with Eq. (2).

1 $${I}_{i}=\frac{1}{n}{\sum }_{j=1}^{n}\frac{{A}_{j}}{{Th}_{j}}$$

where *I*_*i*_ is the value of index *i*, *n* is the number of indicators classified in index *i*, *A*_*j*_ is the experimental value of indicator *j*, and *Th*_*j*_ is the arithmetic mean of all experimental values of indicator *j*.

2 $${SQ}_{z}=\frac{{sin72^\circ (I}_{ms}\times {I}_{ma}+{I}_{ma}\times {I}_{mi}+{I}_{mi}\times {I}_{ea}+{I}_{ea}\times {I}_{ps}+{I}_{ps}\times {I}_{ms})}{2}$$

where *SQ*_*z*_ is the value of soil quality for treatment *z. I*_*ms*_, *I*_*ma*_, *I*_*mi*_, *I*_*ea*_, and *I*_*ps*_ are five segments that have the same length as the value of I_ms_, I_ma_, I_mi_, I_ea_, and I_ps_.

The sustainability index (SI) does not consider the unevenness of soil indices and adopts the condition that soil qualities are the same. However, the soil indices of the different treatments exhibit a difference according to Eq. (2). Therefore, this study created the coefficient of unevenness (*C*_*u*_) calculated according with Eq. (3). A low value of Cu means high evenness in soil capacity and good soil quality under the same SQ value.

3 $${C}_{u}=\frac{\sqrt{\left({I}_{ms}-{M}^{2}\right)+\left({I}_{ma}-{M}^{2}\right)+\left({I}_{mi}-{M}^{2}\right)+\left({I}_{ea}-{M}^{2}\right)+({I}_{ps}-{M}^{2})}}{5}$$

where *M* is the arithmetic mean of the soil indices.

### Data processing and statistical analyses

ANOVA and Tukey’s HSD post hoc tests (p < 0.05) were performed to compare the differences in soil physical and chemical properties and enzyme activities among the different treatments (SPSS, version 19.0, IBM, Armonk, NY, USA), and SI was used to assess the soil quality and change rules.

### Statements

WE statement that this study complies with relevant institutional, national, and international guidelines and legislation, and permissions for collecting the plant and seed specimens has been obtained.

## Supplementary Information

Supplementary Information.

## Data Availability

The datasets used and/or analysed during the current study available from the corresponding author on reasonable request.
